# Active Antimicrobial Packaging Systems: Mechanisms of Microbial Control and Applications in Food Preservation

**DOI:** 10.3390/biology15040325

**Published:** 2026-02-12

**Authors:** Esteban Pérez, Esther Sanjuán, Miroslav Jůzl, António Raposo, Ariana Saraiva, José Raduan Jaber, Conrado Carrascosa

**Affiliations:** 1 Department of Animal Pathology and Production, Bromatology and Food Technology, Faculty of Veterinary, Universidad de Las Palmas de Gran Canaria, Trasmontaña s/n, 35413 Arucas, Spain; esther.sanjuan@ulpgc.es; 2 Department of Food Technology, Faculty of AgriSciences, Mendel University in Brno, 613 00 Brno, Czech Republic; miroslav.juzl@mendelu.cz; 3 CBIOS (Research Center for Biosciences and Health Technologies), ECTS (School of Health Sciences and Technologies), Lusófona University, Campo Grande 376, 1749-024 Lisboa, Portugal; antonio.raposo@ulusofona.pt; 4 Research in Veterinary Medicine (I-MVET), Faculty of Veterinary Medicine, Lisbon University Centre, Lusófona University, Campo Grande 376, 1749-024 Lisboa, Portugal; ariana.saraiva@ulusofona.pt; 5 Veterinary and Animal Research Centre (CECAV), Faculty of Veterinary Medicine, Lisbon University Centre, Lusófona University, Campo Grande 376, 1749-024 Lisboa, Portugal; 6 Department of Morphology, Faculty of Veterinary, Universidad de Las Palmas de Gran Canaria, Trasmontaña s/n, 35413 Arucas, Spain; joseraduan.jaber@ulpgc.es

**Keywords:** microbial spoilage, antimicrobial packaging, foodborne pathogens, microbial ecology, mechanisms of microbial inhibition

## Abstract

Microbial spoilage and foodborne pathogens remain central challenges in food safety, driven by the metabolic resilience and ecological adaptability of bacteria, yeasts, and molds across diverse food matrices. Active antimicrobial packaging has emerged as a biologically informed strategy that directly targets microbial physiology through controlled release or contact-mediated mechanisms. These systems employ natural antimicrobials, bacteriocins, essential oils, and metal nanoparticles to disrupt cell membranes, inhibit enzymatic pathways, generate reactive oxygen species, or interfere with quorum sensing, resulting in substantial reductions in microorganisms such as *Listeria monocytogenes*, *Salmonella* spp., *E. coli* O157:H7, *Pseudomonas* spp., *Brochothrix thermosphacta*, and spoilage fungi. In real food environments, these interventions achieve multi-log reductions and attenuate microbial metabolism, though efficacy varies with pH, water activity, fat content, and storage temperature. Oxygen scavengers further reshape microbial ecology by suppressing aerobic spoilage organisms while inadvertently favoring anaerobic competitors. Despite promising outcomes, concerns regarding nanoparticle migration, microbial resistance potential, and matrix-dependent performance highlight the need for deeper microbiological validation. Future progress will require integrative research linking microbial ecology, packaging material science, and mechanistic toxicology. By aligning with microbial behavior at the cellular and ecosystem levels, active antimicrobial packaging represents a powerful, biologically grounded approach to mitigating foodborne risks.

## Simple Summary

A World Health Organization estimate has identified some 200 types of diseases caused by food contaminated with microorganisms (bacteria, viruses, and parasites), of which some 30 species of bacteria are primarily responsible for food poisoning. Microorganisms, such as bacteria, yeasts, and molds, are naturally present in many foods, and while some are harmless, others can cause spoilage or serious illness. Keeping these microorganisms under control is one of the biggest challenges in protecting the safety and freshness of food. Traditional packaging slows their growth but cannot fully prevent them from surviving or spreading. This article examines new types of “active” packaging designed to directly fight harmful microorganisms. These smart materials can release natural substances from plants, helpful proteins, or very small particles that weaken or damage microbes before they can grow. Some systems also remove oxygen from the package, which stops many microorganisms from multiplying. Research using real foods shows that these technologies can greatly reduce the presence of harmful microbes and extend the life of products such as meat, dairy, fruits, and vegetables. However, more work is needed to be sure that these materials are completely safe for people and the environment and that they perform well in all food types. By understanding how microorganisms behave in packaged foods, scientists can develop better tools to protect consumers, reduce food waste, and support a more sustainable and secure food system.

## 1. Introduction

Food safety is fundamentally shaped by a diverse array of foodborne pathogens and spoilage microorganisms, whose prevalence and impact vary considerably across different food categories. Among the most significant bacterial pathogens are *Salmonella* spp., *L. monocytogenes*, *Escherichia coli* (particularly *E. coli* O157:H7), *Staphylococcus aureus*, and *Campylobacter jejuni*, all of which are frequently implicated in outbreaks associated with meat, dairy, eggs, seafood, fruits, and vegetables [1,2,3,4]. These pathogens possess the ability to survive various food processing and storage conditions. *L. monocytogenes*, for example, demonstrates remarkable resistance to environmental stresses and can persist in food production environments through biofilm formation [5,6].

Beyond these primary pathogens, spore-forming bacteria such as *Bacillus cereus* and *Clostridium* spp. present particular challenges in both animal- and plant-based foods, as their spores can survive thermal processing and subsequently germinate under favorable conditions [7,8]. Additionally, *Cronobacter sakazakii* represents a notable hazard in powdered infant formula and low-moisture foods, while *Vibrio parahaemolyticus* remains a key concern in seafood products [3,9].

While spoilage microorganisms are not always pathogenic, they significantly impact food quality and shelf life. *Pseudomonas* spp. constitute the dominant spoilage agents in raw and processed meats, dairy, and seafood, producing proteases and lipases that degrade food even after pasteurization [10,11]. Fungi, including molds such as *Aspergillus* and *Fusarium,* along with yeasts like *Zygosaccharomyces*, are especially problematic in bakery products, fruits, vegetables, and low-water-activity foods, where they can also produce mycotoxins that pose additional health risks [12,13]. In dairy products and plant-based analogs, high water activity and near-neutral pH further facilitate the growth of both spoilage bacteria and fungi.

The risk of contamination and spoilage is modulated by intrinsic food properties such as pH and water activity, as well as by processing methods and storage conditions. Minimally processed and ready-to-eat foods are particularly susceptible to both pathogenic and spoilage microorganisms due to reduced thermal treatments and extended shelf life requirements [1,8]. Furthermore, the emergence of antimicrobial resistance among foodborne pathogens, as observed in *E. coli* and *Salmonella* strains in meats and vegetables, exacerbates food safety challenges [4].

The main pathogens and spoilage microorganisms associated with each food category are summarized in Table 1.

Food safety across all categories is shaped by the persistent presence of key bacterial pathogens and a wide range of spoilage organisms, whose prevalence varies with food type, processing conditions, and storage environments. Ensuring both safety and quality therefore depends on coordinated strategies that address the risks posed by pathogenic as well as spoilage microbes [1,3,8,10,11,12].

### 1.1. Microbial Challenges Associated with Modified Atmosphere Packaging and Vacuum Packaging

Traditional food packaging methods such as modified atmosphere packaging (MAP) and vacuum packaging (VP) are widely employed to inhibit spoilage and pathogenic microorganisms. However, these technologies present several critical limitations that can compromise food safety and quality.

One major limitation is the selective inhibition of microorganisms. While high concentrations of CO_2_ in MAP can effectively suppress Gram-negative spoilage bacteria and Enterobacteriaceae, they often allow the proliferation of lactic acid bacteria (LAB) and certain facultative anaerobes, which can become dominant and contribute to spoilage or off flavors as storage progresses. In fish and meat products, for instance, LAB thrive under high CO_2_ and low O_2_ conditions, sometimes exceeding spoilage thresholds even when other bacteria are suppressed [14,15]. Similarly, VP, by removing oxygen, inhibits aerobic bacteria but can inadvertently favor the growth of anaerobic or facultative anaerobic pathogens such as *Clostridium botulinum* and *Listeria monocytogenes*, especially when temperature control is inadequate [16,17].

Another significant challenge is the dependence on strict temperature control. The efficacy of MAP and VP is highly contingent on maintaining low storage temperatures. If temperature abuse occurs, the risk of outgrowth and toxin production by psychrotrophic pathogens, particularly *C. botulinum*, increases substantially, as these organisms are not effectively inhibited by the absence of oxygen alone [16,17,18].

Packaging material properties also play a crucial role in determining effectiveness. The permeability of packaging films to gases can lead to gradual changes in the internal atmosphere due to gas diffusion, microbial respiration, or package defects. This can result in the loss of the intended protective environment, thereby reducing the effectiveness of microbial inhibition and potentially compromising food safety [16,19].

Furthermore, MAP and VP do not address all spoilage mechanisms. While they can slow microbial growth, they may not sufficiently prevent enzymatic or oxidative spoilage, and in some cases, MAP can even accelerate undesirable changes such as texture loss or increased drip in certain products like fish and seafood [15,20]. Additionally, the use of high CO_2_ concentrations, while bacteriostatic, can negatively impact sensory qualities, including color and flavor, and may not be suitable for all food types [21,22].

Finally, operational and safety concerns persist. The requirement for skilled staff, high initial costs, and the potential for chemical and microbial hazards associated with improper gas mixtures or packaging failures are practical limitations that can hinder the consistent effectiveness of these technologies [16,23].

These limitations are summarized in Table 2.

Although both MAP and VP are valuable for extending shelf life, their limitations (particularly selective microbial inhibition, temperature dependence, and the need for reliable package integrity) call for careful management and, increasingly, the integration of advanced or active packaging solutions to ensure robust food safety [15,16,17,19].

### 1.2. Active Food Packaging: Definition and Recent Evolution

Active food packaging is defined as a packaging system that deliberately incorporates components designed to release or absorb substances into or from the packaged food or the surrounding environment, with the aim of extending shelf life or maintaining or improving food quality and safety. Unlike traditional packaging, which acts as a passive barrier, active packaging interacts dynamically with the food or its headspace, providing functionalities such as oxygen scavenging, moisture absorption, antimicrobial activity, and antioxidant release [24,25,26]. According to European Regulation 450/2009/EC [27], active materials are specifically intended to modify the condition of packaged food by controlling the transfer of substances, thereby preventing microbial and chemical contamination and preserving sensory attributes [26].

Over the past decade, active packaging technology has undergone a significant transformation, driven by advances in materials science, sustainability concerns, and consumer demand for minimally processed foods. The integration of natural bioactive compounds (such as essential oils, plant extracts, and phenolic compounds) has emerged as a major trend, offering both antioxidant and antimicrobial properties while addressing concerns over synthetic additives. Encapsulation techniques and polymer blending have been increasingly adopted to control the release of these volatile or sensitive compounds, improving their stability and minimizing sensory impacts on food [26,28,29].

The use of bio-based and biodegradable polymers, including chitosan, starch, and polylactic acid, has expanded considerably, supporting the development of environmentally friendly packaging systems that align with circular economy principles [29,30]. Nanotechnology has also played a pivotal role in this evolution, with nano- and microencapsulation enhancing the controlled release and efficacy of active agents, while nanomaterials have improved the mechanical and barrier properties of packaging films [31,32]. Electrospinning and other advanced fabrication methods have enabled the creation of nanofiber-based active films with high surface area and tunable release profiles [33]. Furthermore, the synergy between active and intelligent packaging (where active systems preserve food and intelligent systems monitor quality) has led to the emergence of multifunctional “smart” packaging solutions [24,34]. Commercial applications now include antimicrobial sachets for meat, essential oil-based pads for produce, and oxygen scavenging films for bakery products [29,35]. Despite these advances, challenges remain regarding regulatory approval, consumer acceptance, and the scalability of new materials and technologies. Nevertheless, the last decade has seen active packaging evolve from a niche innovation to a central strategy in sustainable food preservation [24,26,29].

The key developments in active food packaging over the last decade are outlined in Table 3.

In summary, active food packaging has evolved into a sophisticated, multifunctional technology that not only preserves food but also addresses sustainability and consumer health, with rapid innovation in natural compounds, encapsulation, and smart materials [24,26,29,31].

This review was conducted following a structured narrative approach. Scientific literature was retrieved from major bibliographic databases, including Web of Science, Scopus, and PubMed. Searches were performed using combinations of keywords such as active packaging, antimicrobial packaging, foodborne pathogens, spoilage microorganisms, essential oils, bacteriocins, nanocomposites, and real food systems.

The primary focus was placed on peer-reviewed articles published predominantly between 2020 and 2025, while seminal earlier studies were included where necessary to provide historical context. Reviews, experimental studies, and selected regulatory reports were included to ensure comprehensive coverage of technological, microbiological, and safety-related aspects.

## 2. Overview of Microbial Challenges in Packaged Foods

### 2.1. Microbial Growth in Packaged Foods: Matrix-Specific Dynamics and Influencing Factors

Microbial growth in packaged foods is a complex process shaped by the intrinsic properties of the food matrix (such as pH, water activity, fat, and protein content) as well as extrinsic factors like storage temperature, packaging atmosphere, and humidity. These variables interact to determine both the rate and type of microbial proliferation across different food categories, including meat, fish, dairy, and fruits.

### 2.2. Microbial Growth Across Food Matrices

In meat and fish products, high water activity (aw) and near-neutral pH create favorable conditions for rapid microbial growth, particularly for spoilage bacteria such as *Pseudomonas*, *Brochothrix*, and LAB [12,38,39,40,41]. In vacuum-packed and modified-atmosphere packaged meats, LAB often become dominant during storage, while *Pseudomonas* and *Serratia* are prevalent in aerobic conditions or at higher pH [40,42,43]. Fish, with its high moisture and protein content, is especially susceptible to spoilage by psychrotrophic bacteria, which thrive at refrigeration temperatures [38,39].

In dairy products, the presence of lactose and proteins supports the growth of both spoilage and fermentative microbes, with pH and aw again playing central roles [12,44]. Fruits, while generally more acidic, can still support the growth of yeasts and molds, especially when aw is elevated [12,44].

### 2.3. Influence of pH, Water Activity, Fat, and Proteins

pH is a critical determinant of microbial ecology in foods. Most spoilage bacteria prefer near-neutral pH, while acidic environments inhibit bacterial growth but may allow acid-tolerant yeasts and molds to proliferate [12,38,44]. Water activity is equally important: higher aw values facilitate faster microbial proliferation, as seen in fresh meats and fish, whereas dried or high-sugar foods with low aw are more resistant to spoilage [44,45,46,47]. In high-solid matrices, not just the amount but the mobility of water (quantified as water usability) can more accurately predict microbial growth, as protein networks can sequester water and slow spoilage [44,45,46].

Fat and protein content also modulate microbial behavior. High protein levels provide abundant nutrients for bacteria, while fat can influence the types of spoilage organisms and the metabolic pathways they employ, such as shifting from protein to carbohydrate metabolism under certain packaging atmospheres [41,48]. In dairy and meat products, microbial degradation of proteins leads to off odors and textural defects, while fat-associated reactions (e.g., lipid-rich regions) further contribute to quality deterioration during storage [12].

### 2.4. Role of Storage Conditions

Storage temperature is one of the most influential extrinsic factors affecting microbial growth. Lower temperatures generally slow microbial growth, but psychrotrophic bacteria can still proliferate in refrigerated meats and fish [39,40,41]. Temperature fluctuations accelerate spoilage, particularly in fish, by favoring the growth of spoilage bacteria like *Serratia* and *Carnobacterium* [22].

Packaging atmosphere also shapes microbial communities: MAP with elevated CO_2_ can suppress aerobic spoilage bacteria and favor LAB, thereby extending shelf life [15,39,41,42]. Vacuum packaging similarly limits oxygen, promoting LAB dominance and slowing the growth of aerobic spoilage organisms [40,42,49].

The main factors influencing microbial growth across different food matrices are presented in Table 4.

In traditional fermented products (cheeses, cured sausages, sauerkraut, kimchi), sensory quality and safety depend on a dynamic microbial succession, led mainly by lactic acid bacteria (LAB), yeasts and, occasionally, beneficial molds. The introduction of active technologies can alter this balance. For artisanal or traditionally fermented foods, active packaging must be carefully tailored. Ideally, it should activate after fermentation, during distribution or storage. Also, for authentic traditional labeling, regulators (such as EU PDO products) may restrict such packaging interventions. Overall, active packaging can interfere with natural fermentation by disrupting microbial ecology or environmental conditions essential for fermentation. However, if properly designed (for post-fermentation quality control rather than microbial suppression during fermentation) it can complement traditional processes rather than hinder them [50,51].

## 3. Active Packaging Technologies and Microbial Control

### 3.1. Antimicrobial-Releasing Systems

**Figure 1:**
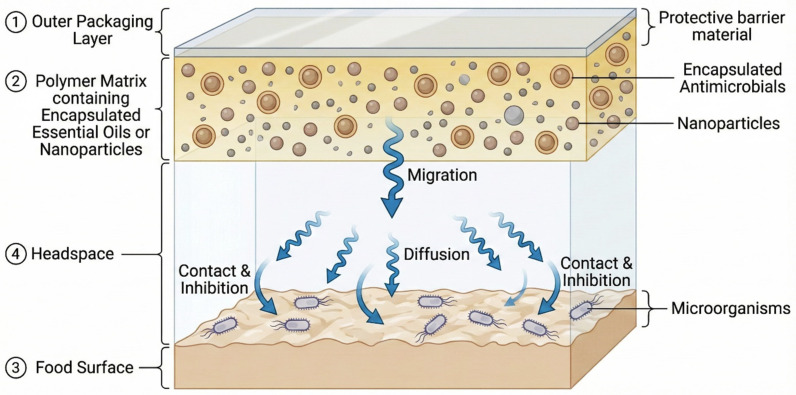
Schematic representation of controlled antimicrobial release from active packaging to food.

Antimicrobial-releasing systems function through the controlled migration of bioactive compounds from the packaging matrix to the food surface or headspace (Figure 1). This process is governed by diffusion kinetics, which depend on polymer properties, antimicrobial characteristics, and environmental conditions. The released agents can act either through direct contact with the food surface or via vapor phase distribution in the package headspace.

#### Effectiveness of Essential Oils, Phenolic Extracts, and Plant-Derived Antimicrobials in Active Packaging Films

Natural antimicrobial agents incorporated into active packaging films have demonstrated substantial efficacy against foodborne pathogens and spoilage organisms in real food systems. Clove essential oil (CEO) incorporated into PET/PP films with a pullulan coating significantly inhibits the growth of foodborne pathogens such as *E. coli*, *S. enteritidis*, *S. aureus*, and *L. monocytogenes* on spinach leaves. The antimicrobial effect is dose-dependent, and the films maintain transparency and sensory acceptability, making them suitable for leafy greens [52].

Basil essential oil, when microencapsulated and grafted onto chitosan-based edible films, demonstrates strong inhibitory effects against Gram-positive bacteria. When these films are used to wrap cooked ham, they control bacterial growth and prevent pH increase during storage, effectively extending shelf life [53]. Similarly, chitosan films with *Plectranthus amboinicus* essential oil show improved barrier properties and promising antimicrobial activity against food pathogens, supporting their use for shelf life extension in various foods [54].

Oregano essential oil in alginate-based edible films completely inhibits the growth of tested bacteria and fungi, including common spoilage organisms, and shows high antioxidant activity. These films are proposed as biodegradable alternatives for food packaging with potential for broad application [55]. Chitosan films containing oregano and thyme essential oils, when applied to beef, inhibit spoilage bacteria and pathogens such as *S. aureus*, *E. coli* O157:H7, and Salmonella Typhimurium, prolonging shelf life by approximately 10 days while maintaining sensory quality [56].

Carvacrol-loaded halloysite nanotubes in low-density polyethylene films provide excellent antifungal efficacy against Penicillium commune in bread and cherries, increasing bread shelf life by over 73% and cherry salability by 40%, even after 20 months of storage [57]. Phenolic-rich plant extracts, when incorporated into packaging, also inhibit a broad spectrum of bacteria and fungi, though their effectiveness can be reduced in complex food matrices compared to in vitro conditions. Encapsulation and association with polymers enhance their stability and controlled release, improving their practical application in real foods [58].

### 3.2. Contact-Active Films and Antimicrobial Surfaces

#### Chitosan-Based Films and Metal Nanoparticles: Mechanisms and Evidence for Microbial Inhibition

Chitosan-based films exhibit intrinsic antimicrobial activity due to their polycationic nature, which enables interaction with negatively charged microbial cell membranes, leading to increased membrane permeability and leakage of intracellular contents. This mechanism is further enhanced when chitosan is combined with metal nanoparticles such as ZnO, Ag, and Cu, which introduce additional modes of microbial inhibition [59,60].

The incorporation of ZnO nanoparticles into chitosan films significantly improves antibacterial efficacy. ZnO acts through multiple mechanisms: it releases Zn^2+^ ions that disrupt bacterial enzyme systems, generates reactive oxygen species (ROS) under light exposure, and interacts directly with microbial membranes, causing structural damage and cell death. Chitosan-ZnO films achieved 90% growth inhibition of *Listeria monocytogenes* and demonstrated bactericidal effects against Staphylococcus aureus and Campylobacter jejuni, with smaller ZnO particles showing greater activity due to increased surface area and reactivity [59]. In real food applications, chitosan-ZnO nanocomposite films have been used to wrap chicken filet and cheese, effectively inhibiting spoilage bacteria and maintaining food quality during storage [59,60]. Similarly, chitosan films with ZnO nanoparticles extended the shelf life of guava fruit by one week, reducing microbial load and spoilage compared to unpackaged controls [61].

Silver nanoparticles (AgNPs) embedded in chitosan matrices provide potent antimicrobial action at low concentrations. AgNPs interact with bacterial cell membranes and DNA, disrupting cellular processes and leading to cell death. The synergistic effect of Ag and Zn ions in composite films further amplifies antimicrobial activity. Gelatin/chitosan films containing AgNPs and ZIF-8 (a zinc-based metal–organic framework) eliminated *E. coli* and *S. aureus* within three hours, demonstrating rapid and effective pathogen control in perishable fruits [62]. The release behavior of silver ions from chitosan-based films is crucial, as sustained release ensures prolonged antimicrobial protection in food systems [63,64].

Copper oxide nanoparticles (CuO NPs) in chitosan films also display strong antimicrobial activity, primarily through the generation of ROS and disruption of microbial metabolic pathways. Chitosan-CuO nanocomposite films showed the highest inhibition zones against Enterobacter cloacae, Salmonella, *S. aureus*, and Campylobacter, and when used to package guava, these films minimized microbial load and spoilage, outperforming both control chitosan films and unpackaged fruit [61]. The effectiveness of these nanocomposite films extends beyond laboratory conditions to practical food packaging scenarios, where chitosan-based films with ZnO, Ag, or Cu nanoparticles have been shown to inhibit a broad spectrum of foodborne pathogens and spoilage organisms, extend shelf life, and preserve sensory and physicochemical qualities in products such as meat, cheese, fruits, and vegetables [59,60,61,62,63].

**Figure 2:**
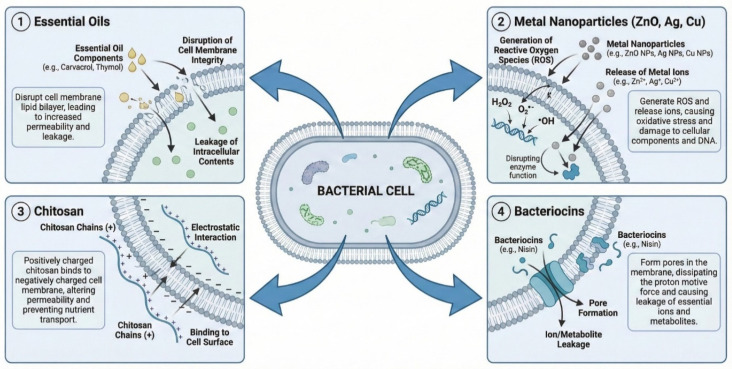
Antimicrobial mechanisms of active packaging agents against microbial cells.

These diverse antimicrobial mechanisms are illustrated in Figure 2. As depicted, chitosan disrupts microbial membranes through electrostatic interactions, while metal nanoparticles act via ion release and ROS generation. Essential oils compromise membrane integrity, and bacteriocins form membrane pores. This multi-target approach explains the broad-spectrum efficacy of active packaging systems.

### 3.3. Oxygen Scavengers

Oxygen scavengers in food packaging rapidly reduce residual oxygen, often to below 100 ppm, which directly suppresses the growth of aerobic spoilage bacteria such as *Pseudomonas* spp. and Acinetobacter–Moraxella spp. in seafood and meat, thereby extending shelf life and maintaining product quality [65,66]. This shift in the packaging environment alters the dominant microbial flora, favoring Gram-positive bacteria like *Brochothrix thermosphacta* and LAB, which are less associated with spoilage odors and off flavors [41,65]. In cooked sausages and bison meat, oxygen scavengers have been shown to significantly inhibit the development of aerobic microflora and reduce oxidation, confirming their efficacy in real food systems [66,67].

The mechanism underlying this effect is the deprivation of oxygen required for the metabolism of obligate aerobes, which suppresses their growth and the associated spoilage pathways, such as proteolysis and oxidative rancidity [41,65,66]. Predictive models and metagenomic analyses further confirm that oxygen scavengers and MAP consistently reduce the growth rates and metabolic activity of aerobic spoilage organisms, leading to extended shelf life [41,68].

However, the creation of anaerobic or microaerophilic conditions introduces a potential risk: the selective advantage for anaerobic pathogens, most notably *Clostridium botulinum*. In the absence of oxygen, *C. botulinum* can germinate and produce botulinum neurotoxin if other hurdles (such as low pH, salt, or preservatives) are not present [69,70]. In uncured shredded pork and turkey under reduced oxygen, botulinum toxin was detected in control samples but not in those treated with a combination of cultured dextrose–vinegar–rosemary extract and citric acid, which inhibited both *L. monocytogenes* and *C. botulinum* for up to 12 weeks, even under mild temperature abuse [70]. This demonstrates that while oxygen scavengers are effective against aerobic spoilage, additional hurdles are essential to prevent the outgrowth and toxin production of anaerobic pathogens.

### 3.4. Other Active Systems

Antioxidant-releasing films, moisture absorbers, and CO_2_ emitters have each demonstrated efficacy in reducing microbial spoilage across a range of food products. Cellulose-based films enhanced with antimicrobial and antioxidant agents, such as chitosan and cinnamaldehyde, have shown strong antibacterial activity against *Staphylococcus aureus* and *E. coli*, effectively suppressing microbial growth and extending the shelf life of fresh produce like strawberries by reducing decay and maintaining firmness [71]. Similarly, zein/poly (lactic acid) films loaded with essential oils such as carvacrol and cinnamaldehyde achieved over 99% inhibition of *E. coli* and *S. aureus*, highlighting the synergistic effect of natural antioxidants and antimicrobials in active packaging [72].

Moisture absorbers, such as active absorbent pads in MAP, have been shown to significantly lower total viable counts and specific spoilage organisms in omega-3-enriched rabbit meat, while also reducing lipid oxidation and preserving sensory attributes over extended storage periods [73]. Triple-layer nanofiber pads with directional liquid absorption and controlled chlorine dioxide release further exemplify this approach, as they prevent produce from being wetted, absorb excess moisture, and release antimicrobial agents in response to moisture, thereby inhibiting *E. coli*, *S. aureus*, and *Aspergillus niger* [74].

CO_2_ emitters and high-CO_2_ MAP systems are effective in inhibiting the growth of aerobic spoilage bacteria. Packaging chicken filets with 60% CO_2_/40% N_2_ resulted in significant growth inhibition of spoilage isolates compared to 100% N_2_ and also reduced the production of off odors associated with spoilage [75]. In smoked chicken legs, MAP with 60% or 100% CO_2_ delayed bacterial spoilage and extended shelf life, with *Carnobacterium* and *Pseudomonas* as the predominant genera under these conditions [76]. Biodegradable sachets combining water absorption and CO_2_ emission, such as PBAT/TPS-ZnO systems, have also demonstrated over 99% reduction in Gram-positive and Gram-negative bacteria, while maintaining optimal moisture and gas balance in the package headspace [77].

Incorporating natural extracts, essential oils, and nanoparticles into biopolymer films further enhances antimicrobial and antioxidant activity, as seen with chitosan/rosemary oil films that inhibit *Listeria monocytogenes* and *E. coli* and pectin/gelatin films with tannic acid and silver nanoparticles that extend the shelf life of strawberries by blocking UV and suppressing bacterial growth [32,78]. These strategies collectively illustrate the multifaceted benefits of active packaging in controlling microbial spoilage and preserving food quality.

### 3.5. Comparative Synthesis of Active Antimicrobial Packaging Technologies

Taken together, the active antimicrobial packaging strategies described above differ substantially in terms of antimicrobial performance, technological maturity, economic feasibility, food matrix compatibility, and environmental sustainability. While each approach offers specific advantages, their practical implementation requires a comparative, application-oriented assessment rather than an isolated evaluation of individual technologies.

From an application-oriented perspective, comparing active antimicrobial packaging systems is essential for their effective translation into practice. These technologies differ significantly in antimicrobial performance, economic viability, scalability, food matrix compatibility, and environmental sustainability.

Natural systems based on essential oils, phenolic compounds, and bacteriocins generally provide moderate to high antimicrobial efficacy, particularly against Gram-positive bacteria and spoilage fungi, while offering advantages in consumer acceptance, regulatory compliance, and compatibility with biodegradable polymers. However, their use is constrained by volatility, sensory interactions, and matrix-dependent effectiveness.

In contrast, nanocomposite systems incorporating metal nanoparticles (e.g., Ag, ZnO, CuO) exhibit strong and broad-spectrum antimicrobial activity but are associated with higher costs, regulatory complexity, and unresolved concerns regarding migration, toxicity, and environmental impact, limiting their application to high-value or high-risk food products.

Oxygen scavengers and gas-modulating systems represent technologically mature, scalable, and cost-effective solutions, primarily effective against aerobic spoilage and oxidative degradation, though they often require complementary hurdles to mitigate the risk of anaerobic pathogens.

Overall, the selection of active antimicrobial packaging technologies should rely on a balanced assessment of antimicrobial efficacy, economic feasibility, environmental footprint, and regulatory practicality, emphasizing application-specific designs aligned with food matrix characteristics, shelf life requirements, and sustainability goals.

## 4. Microbial Responses in Real Foods

### 4.1. Spoilage Microorganisms

Natural antimicrobial active packaging demonstrates broad efficacy against key spoilage microorganisms, though responses vary by species and antimicrobial agent. *Pseudomonas* spp. and *Brochothrix thermosphacta*, both major meat spoilage bacteria, are notably sensitive to plant-derived compounds such as thymol, carvacrol, cinnamaldehyde, and phenyllactic acid. In vitro, these agents achieve IC_50_ values for spoilage bacteria in the range of 0.35–0.75 mM, with *Shewanella putrefaciens* showing the highest sensitivity among tested spoilage organisms [79,80]. In real food systems, phenyllactic acid incorporated into packaging or applied with MAP significantly reduces the growth and metabolic activity of *Pseudomonas lundensis* and *Brochothrix thermosphacta*, as evidenced by lower bacterial counts and reduced spoilage indicators in pork loins [81].

**Figure 3:**
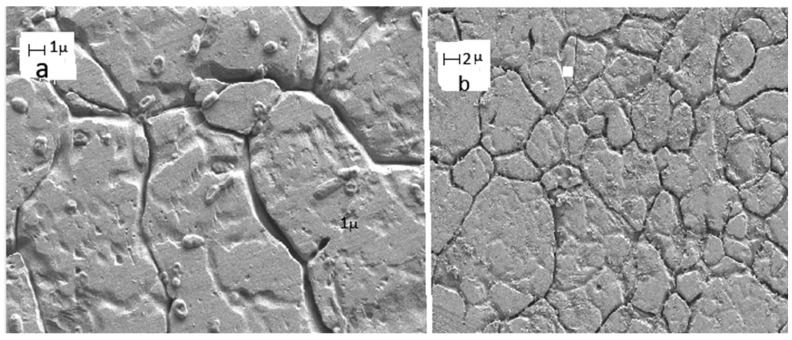
Comparation of SEM samples of *Pseudomonas aeruginosa*. (**a**) Bacteria biofilm with essential oil of thyme. (**b**) Bacteria biofilm without treatment of essential oil of thyme. The SEM image in the left-hand show bacteria with clear damage in the surface. On the right-hand side, the image prepared without thyme show the presence of biofilms and intact bacteria.

Structural damage to microbial cells and disruption of biofilm architecture have been directly observed in several studies using microscopic techniques. As illustrated in Figure 3, exposure to thyme essential oil results in pronounced surface damage and loss of cellular integrity in *Pseudomonas aeruginosa*, whereas untreated cells exhibit intact morphology and dense biofilm formation. These observations support the proposed membrane-targeting mechanisms underlying the antimicrobial efficacy of essential oil-based active packaging systems.

Overall, natural antimicrobial active packaging (using essential oils, plant extracts, chitosan, and protective cultures) consistently inhibits the growth and spoilage activity of *Pseudomonas*, *Shewanella*, *Brochothrix thermosphacta*, yeasts, and molds in real food matrices, with efficacy dependent on the specific agent, food type, and storage conditions [79,80,81,82,83,84,85]. The responses of key spoilage microorganisms to natural antimicrobial active packaging are summarized in Table 5.

### 4.2. Foodborne Pathogens

#### Effects on *Listeria monocytogenes*, Salmonella, *E. coli* O157:H7, and Spore-Forming Bacteria

- *Listeria monocytogenes*: Natural antimicrobial active packaging (especially those incorporating bacteriocins (e.g., nisin, plantaricin W), essential oils, and metal nanoparticles) consistently demonstrates significant inhibition of *L. monocytogenes* in real food systems. Nisin-loaded films and coatings applied to ready-to-eat ham, cheese, and sausages have achieved complete inhibition or reductions of 4–6 log CFU/g during refrigerated storage, with shelf life extension and no adverse sensory effects [91,92,93,94]. Chitosan-based and cellulose nanofibril films with nisin or enterocin also show strong anti-listerial activity in meat and dairy products [91,94]. Essential oil-coated papers and biopolymer films (e.g., with cinnamon, clove, or carvacrol) reduce Listeria counts in raw meat and cheese, with reductions of 1–3 log CFU/g reported [95,96]. Copper-loaded carbon nanoparticles and lysozyme-activated films further enhance inhibition, especially in acidic foods [92,93].
- *Salmonella* spp.: Active packaging with natural antimicrobials such as caprylic acid, essential oils, and bacteriocins effectively reduces Salmonella in meat and produce. PLA films with 4.8% caprylic acid reduced *S. typhimurium* and *S. enteritidis* by up to 2.6 log CFU/g on chicken and beef without affecting quality [97].
- Diacetyl-based sachets in beef packaging achieved up to 77% inhibition of *Salmonella enterica* during refrigerated storage [98]. Essential oil-infused films and coatings (e.g., oregano, garlic, carvacrol) have demonstrated 1–3 log reductions in Salmonella on meat and cheese, with some studies reporting complete inactivation in whey protein matrices [95,96]. Bacteriophage-based packaging also targets Salmonella with high specificity and efficacy [88].
- *E. coli* O157:H7: Natural antimicrobial packaging, including essential oils, bacteriocins, and phage-based films, is effective against *E. coli* O157:H7 in real foods. Carvacrol- and clove oil-enriched films inhibit *E. coli* biofilms by over 80% and reduce planktonic cells by several log cycles [99,100]. Composite phage nanofiber films with D-phenylalanine achieve > 99.7% reduction in *E. coli* O157:H7 and >99.99% inhibition of biofilm formation on beef and produce during storage [101]. Chitosan/curcumin coatings and essential oil-coated papers also reduce *E. coli* O157:H7 on food contact surfaces and raw meat [33,95].
- Spore-forming bacteria: Bacteriophage-based packaging and metal nanoparticles (e.g., ZnO, Ag, Cu) incorporated into biopolymer films show activity against spore-forming bacteria such as *Bacillus* spp. and *Clostridium botulinum*. Chitosan-based and cellulose-based films with essential oils or metal nanoparticles inhibit Bacillus cereus and other spore-formers in meat and dairy products, though efficacy may depend on the food matrix and environmental conditions [84,102].
- A summary of documented antimicrobial effects against major foodborne pathogens in real food systems is provided in Table 6.

### 4.3. Modulating Factors

The antimicrobial effectiveness of active packaging is significantly influenced by pH, water activity (a_w_), food composition, and storage temperature. These factors affect both the release of antimicrobial agents from packaging and their activity against microorganisms.

- pH: pH can alter the release rate and activity of antimicrobial agents. Some films, such as those containing anthocyanins or essential oils, show pH-responsive release, with higher or lower pH triggering more rapid diffusion of the active compound and enhancing antimicrobial action [104,105]. The antimicrobial activity of certain agents (e.g., nisin, silver zeolite) is also directly affected by pH, with optimal activity often observed in acidic environments [106,107]. Films with oregano essential oil or phenolic acids are more effective at low pH, which is typical for many food products [108,109].
- a_w_: a_w_ influences both the migration of antimicrobials from packaging and microbial growth. Lower a_w_ can reduce the diffusion of active compounds and limit microbial proliferation but may also decrease the release rate of some agents from the film matrix [107,109]. In gelatin/chitosan films, reduced a_w_ (achieved by adding sucrose or glycerol) decreased both antioxidant and antimicrobial activity, likely due to lower release rates [109].
- Food composition: Food matrix components such as fat and protein content significantly affect antimicrobial partitioning and efficacy. Higher fat content in foods can absorb more hydrophobic antimicrobials (e.g., carvacrol, allyl isothiocyanate), reducing their concentration in the headspace and thus their antimicrobial effectiveness [110,111]. Conversely, higher protein content can retain more volatile antimicrobials in the headspace, potentially increasing their activity [111]. The presence of carbohydrates, amino acids, and other food constituents can also interact with antimicrobials, modifying their release and activity [88,106].
- Storage temperature: Temperature is a critical factor for both the release kinetics of antimicrobials and microbial growth rates. Higher storage temperatures generally increase the diffusion rate of active agents from packaging, enhancing antimicrobial effectiveness [102,106,107,112]. For example, lysozyme release from PLA films was faster at 10 °C than at 4 °C, resulting in greater inhibition *L. monocytogenes* in smoothies [112]. However, lower temperatures may reduce the overall reduction rate of bacteria due to slower agent release and reduced microbial metabolism [107,113]. The effects of these modulating factors on antimicrobial packaging efficacy are summarized in Table 7.

Food packaging materials must ensure consumer safety and guarantee the safety and organoleptic properties of the food. The nanoparticles contained in the packaging must not interfere negatively with the food. Most studies on the addition of nanoparticles to food packaging [114,115] examine the influence of the pH and temperature of nanoparticles, such as Ag, ZnO, and Cd, on the bactericidal capacity of these compounds. However, the behavior of these particles, such as ZnO, was studied by Omar et al. [116] in a pH range of 3 to 10, finding that the state of the solution influenced the zeta potential and aggregate size stability. The most stable pH region was 7.7, with two unstable regions detected: 4.4 < pH < 6.4 and 8.3 < pH < 10.5. Ag is another nanoparticle used in packaging with a bactericidal effect. Morais et al. [117] conducted a review study on the use of Ag nanoparticles in food packaging to take advantage of its bactericidal effect in order to extend the shelf life of products [118,119]. However, there is a possible effect of Ag migration from the polymer to the food [120].

## 5. Limitations and Safety Considerations

### 5.1. Technological Limitations

Achieving precise, sustained release of antimicrobials remains one of the primary technological challenges. Uncontrolled migration (especially in acidic foods) can lead to loss of functionality and safety risks, as nanoparticles may detach or dissolve from packaging, altering film properties and reducing antimicrobial effectiveness [77]. Additionally, incorporating nanoparticles (e.g., ZnO, Ag) can compromise mechanical, barrier, or thermal properties of packaging, especially in biopolymer films [77,121]. Large-scale, cost-effective production and consumer acceptance remain significant hurdles for commercial adoption [122].

### 5.2. Safety Concerns

Migration of nanoparticles (ZnO, Ag) into food is a major concern, especially under certain conditions (e.g., acidic foods), potentially leading to human exposure and toxicity [77,121,123]. Nanoparticles can cause cytotoxicity, genotoxicity, and bioaccumulation, and their small size increases the risk of absorption and health hazards, with long-term effects not fully understood [121,123,124]. Furthermore, migration of active agents or nanoparticles can alter food taste, odor, or appearance, affecting consumer acceptance [77].

### 5.3. Cost of Implementation

Active and smart food packaging technologies increase industrial costs modestly, as exemplified by smart Tetra Pak packaging, which raises costs slightly (0.0928 EUR per unit) but significantly reduces food waste and climate impact by about 29% [125]. Technologies add processing steps such as incorporating antioxidant layers and sensors, which can increase complexity and production costs [125,126]. Life cycle assessments (LCA) show trade-offs: while packaging impacts rise due to added materials and energy use, overall environmental burdens often decrease because extended shelf life reduces food waste, a major contributor to emissions [125,127,128]. However, scalability and cost-effectiveness remain challenges, especially for bio-based and biodegradable smart materials, which require further optimization for industrial adoption [126,127,129]. Regulatory and consumer acceptance barriers also influence implementation costs and environmental outcomes [130,131]. Overall, the net sustainability benefit depends on balancing increased packaging impacts against food waste reduction gains.

### 5.4. Regulatory Challenges

Regulatory frameworks for nanomaterials in food packaging are still developing, marked by a lack of harmonized global standards and clear risk-assessment protocols. Although authorities such as the EU and FDA have implemented strict migration limits, comprehensive toxicological data for many nanomaterials remain insufficient. Consequently, each new material requires extensive migration and toxicity testing (often involving novel food authorization procedures and specific labeling of nanoparticle content and associated risks) significantly increasing development time and cost [123,124].

## 6. Future Perspectives

To fully realize the potential of active antimicrobial packaging, future research must address several interconnected domains, including real food validation, consumer acceptance, sustainability, and long-term safety, while also embracing emerging smart, responsive, and digitally integrated approaches that extend microbial control beyond static material functions.

### 6.1. Real Food Validation

A significant limitation in current research is the predominance of laboratory-based studies, which do not always reflect the complexities of real food systems. There is a pressing need for studies that evaluate antimicrobial efficacy, migration of active agents, and sensory impacts directly in actual food products under realistic storage and distribution conditions. Many promising materials, such as polysaccharide-based films with natural antimicrobials, have demonstrated efficacy in vitro, but their performance in real foods remains underexplored and requires standardized testing protocols to ensure reliability and comparability across food matrices [29,89].

Consumer acceptance is pivotal for the commercial success of antimicrobial packaging. Research should focus on understanding consumer perceptions, particularly regarding the use of natural versus synthetic antimicrobial agents and any visible or sensory changes in packaged foods. Sensory evaluations have shown that films incorporating essential oils or plant extracts can improve consumer acceptance, but transparency in labeling and communication about safety and benefits is essential to build trust and encourage adoption [20,29].

### 6.2. Sustainability

Sustainability is a core driver in the development of new packaging materials. Future work should prioritize the design and optimization of biodegradable, renewable, and compostable packaging that maintains antimicrobial efficacy and mechanical integrity. The valorization of agro-industrial by-products and the integration of eco-friendly agents are promising strategies that align with circular economy principles and reduce reliance on petrochemical-based plastics [89,132,133,134].

### 6.3. Long-Term Safety

Long-term safety remains a critical concern, especially regarding migration, bioaccumulation, and chronic exposure to antimicrobial agents and nanoparticles. Comprehensive toxicological studies and harmonized regulatory standards are needed to ensure that new materials are safe for consumers and the environment. The potential risks associated with nanomaterials such as ZnO or silver nanoparticles highlight the necessity for case-by-case safety assessments and clear regulatory guidelines [89,121,122].

Beyond these established research priorities, recent advances point toward a new generation of antimicrobial packaging systems that combine responsiveness, sensing, and digital connectivity.

Future antimicrobial food packaging is likely to move from static, additive-based systems toward responsive, information-rich platforms that manage microbial risks in real time, in close connection with actual food behavior and supply chain conditions.

A first trajectory concerns smart responsive release systems that better align antimicrobial delivery with microbial growth dynamics in real foods. Intelligent controlled-release packaging already demonstrates that release triggered by temperature, humidity or volatile metabolites can substantially slow spoilage and enhance safety in perishable products [135]. Extending this approach will require coupling release kinetics to mechanistic models of migration, microbial inactivation, and quality loss for specific matrices such as chilled meat, fish, and fresh-cut produce. Responsive nanomaterials and electrospun biopolymer nanofibers provide high surface area, tunable porosity, and the possibility of multi-step release, which can sustain antimicrobial activity while limiting overall migrant load [102,136]. Importantly, future work must assess how such stimuli-responsive systems perform under fluctuating, non-ideal cold chain conditions, rather than in narrowly controlled laboratory settings [131,135].

In parallel, biosensor-based intelligent packaging is evolving from simple colorimetric indicators toward integrated sensing platforms directly linked to microbial safety endpoints. Current systems employing gas, pH, or volatile amine sensors already correlate well with spoilage and pathogen growth in meat and seafood [137,138]. Incorporating nanomaterial-based sensors and conducting polymer coatings can improve sensitivity to microbial metabolites and biofilm formation while simultaneously providing antimicrobial or antioxidant functions at the surface–food interface [139,140]. For microbial control, the most promising direction is not merely more sensitive sensing but the design of feedback architectures in which biosensor output modulates antimicrobial release or triggers secondary barriers (e.g., headspace modification), creating a closed loop between detection and intervention [135,136].

A third line of development lies in digitally connected packaging, where embedded tags and low-cost sensor nodes communicate with IoT infrastructures. Such systems already enhance traceability and allow remote monitoring of temperature, humidity, and gas composition across distribution chains for meat, fruits, and vegetables [131,138,141]. When combined with cloud analytics and digital twins of products and storage environments, these data can feed predictive microbiology models that estimate remaining shelf life and probability of unsafe microbial loads, supporting dynamic routing, adaptive storage regimes, and more conservative decisions for at-risk batches [142,143,144]. Digital twins also offer a route to virtually test packaging designs and active agents under realistic distribution scenarios before costly in-plant trials.

Realizing these perspectives will require a tighter coupling between materials science, microbiology, and data science. For antimicrobial systems, critical gaps include standardized in situ validation in complex foods, harmonized approaches to nanoparticle and migrant safety assessment, and the development of bio-based matrices that reconcile microbial control with end-of-life sustainability [102,132,136]. For sensor- and IoT-enabled packaging, robust calibration against microbiological criteria, data security, and clear regulatory pathways remain essential preconditions for deployment in safety-critical applications [141,143]. Ultimately, progress will depend less on any single technology than on interoperable platforms in which responsive materials, biosensing, and digital infrastructures are co-designed around concrete microbial hazards and real food use cases.

### 6.4. Commercialization Prospects: Scale-Up, Economics, and Sustainability

Although active antimicrobial packaging is technically mature, only a limited subset of concepts has progressed beyond pilot scale, largely because industrial feasibility, cost, and sustainability benefits must be demonstrated at the level of the full food-packaging system rather than the material alone.

From a scale-up perspective, processes that are compatible with existing converting infrastructure are at a clear advantage. Reviews of antimicrobial-coated films emphasize that gravure, flexography, and industrial roll coating can translate laboratory coatings into continuous manufacturing, whereas electrospinning or complex multilayer assemblies remain difficult to implement at commercial line speeds and widths [145]. Similar conclusions emerge for edible and nano-enabled coatings, where encapsulation and coating steps must be integrated into conventional film extrusion or lamination to avoid prohibitive capital and operating costs [146,147]. Biopolymer-based systems, including chitosan, starch, and cellulose films, show strong technical performance but often suffer from moisture sensitivity, narrow processing windows, and variable raw material quality, all of which complicate industrial standardization and quality control [32,102].

Economic analyses indicate that cost–benefit assessments must account for food savings as well as packaging costs. In a detailed LCA-linked economic study of smart active packaging for Tetra Pak type systems, adding an antioxidant layer and a spoilage sensor marginally increased unit packaging cost but yielded substantial benefits when reduced food waste was considered, leading to improved overall economic performance [125]. A similar life cycle study on active pastry cream bags showed that, when only the empty packages were compared, antimicrobial bags had higher environmental burdens due to the additional coating step; however, once shelf life extension (from 3 to 13 days) and reduced waste were included, the active system decreased environmental impacts by 45–75% across categories [148]. These results suggest that higher material and processing costs can be justified where microbial control measurably reduces returns, spoilage, or safety-driven product withdrawals.

There is growing recognition that commercialization must be framed within life cycle and circular economy logics. Reviews of green active packaging and biodegradable active films stress that environmental gains from bio-based or nano-enabled systems cannot be assumed; they depend on end-of-life options, compatibility with recycling streams, and the balance between added functional layers and avoided food loss [32,149]. The pastry cream case confirms that the dominant contribution to climate impacts lies in the food itself, so even modest extensions in microbiologically safe shelf life can outweigh the added footprint of active layers [148]. Recent work on electrospun biodegradable membranes makes a similar point: the membranes biodegrade rapidly and improve microbial stability, but raw material costs, especially natural actives, currently undermine economic sustainability and would require policy or market incentives to be competitive with commodity plastics [24].

A recurrent theme across techno-economic and LCA-oriented studies is that regulation, safety assessment, and consumer acceptance remain decisive commercial bottlenecks. Comprehensive reviews highlight the need for harmonized migration testing, toxicological evaluation of nanoparticles and bioactives, and clear labeling to support risk communication and trust, particularly when live cultures or nanostructured antimicrobials are used [32,102,146]. At the same time, many active systems with promising in vitro antimicrobial performance still lack validation in realistic logistics chains, formal shelf life modeling, and sensory studies in target foods, limiting the data required for investment decisions and regulatory dossiers [29,148].

### 6.5. Risk Models for Chronic Exposure to Nanoparticles from Food Packaging

Across food-packaging nanotoxicology, risk assessment remains largely framework-driven and screening-level, with few truly quantitative chronic consumer exposure models tailored to nanoparticles in food contact materials (FCMs) or active packaging.

EFSA’s nano risk assessment guidance and the companion Particle-TR document provide the most developed regulatory framework, specifying when nanospecific assessment is needed, how to characterize small-particle fractions, and a tiered approach to exposure and toxicity testing for FCMs, additives, and pesticides [150,151]. These frameworks call for oral exposure assessment that distinguishes dissolved vs. particulate forms, dissolution in gastrointestinal fluids, and, where relevant, additional studies on toxicokinetics, chronic toxicity, and effects on the gut microbiome [150,151,152]. However, they stop short of offering validated quantitative exposure models specific to nanomaterial migration and long-term intake.

In food packaging reviews, migration is recognized as the initiating event for consumer exposure, yet exposure models are mostly conceptual: they couple worst-case or simplified migration estimates with conventional dietary intake assumptions, with limited nanoparticle-specific refinement [153,154,155,156]. Reviews highlight major uncertainties in detecting and quantifying nanoforms in foods, the influence of food matrix and gastrointestinal transformations, and the lack of robust datasets needed to calibrate or validate exposure or in silico toxicity models [153,154,157].

More advanced toxicokinetic and probabilistic approaches are being developed in nearby domains (e.g., nano- and microplastics) using physiologically based kinetic (PBK) models and probability density functions to bridge external exposure and internal doses and to represent particle heterogeneity [158,159]. Conceptual PBK frameworks could, in principle, be adapted to food-packaging nanoparticles, but they require high-quality biodistribution and dissolution data that are currently sparse for most FCM nanomaterials. Life cycle risk assessments for cellulose nanomaterials in food contact applications illustrate how qualitative exposure ranking and high-throughput hazard screening can indicate low risk, yet they also emphasize that long-term low-dose consumer exposure and migration data remain key gaps for more quantitative risk characterization [160,161].

Overall, regulators have structured guidance but not mature, validated chronic exposure models specific to nanoparticles in food packaging. Key limitations include the following: (i) poor analytical capability to distinguish particulate vs. ionic species in foods; (ii) scarce, often short-term oral toxicokinetic and microbiome data; (iii) absence of harmonized chronic exposure scenarios incorporating food–matrix and gastrointestinal transformations; and (iv) limited integration of probabilistic and PBK approaches into routine regulatory practice [150,153,154,156,157,158,159]. As a result, current nano-FCM assessments remain conservative, case-by-case, and heavily reliant on uncertainty analysis rather than well-parameterized chronic risk models.

## 7. Conclusions

Food safety remains a complex challenge shaped by the diverse ecology of foodborne pathogens and spoilage microorganisms, whose behavior is modulated by intrinsic food properties, processing methods, and storage conditions. Traditional packaging approaches such as MAP and VP, while valuable for extending shelf life, present inherent limitations including selective microbial inhibition, temperature dependence, and the potential to favor anaerobic pathogens under inadequate conditions.

Active antimicrobial packaging has emerged as a promising strategy to address these limitations. The incorporation of natural antimicrobial agents (including essential oils, plant extracts, bacteriocins, and chitosan) into packaging films has demonstrated substantial efficacy against both spoilage microorganisms and major foodborne pathogens such as *Listeria monocytogenes*, *Salmonella* spp., and *E. coli* O157:H7 in real food matrices. Similarly, metal nanoparticles and oxygen scavenging systems have shown considerable potential, though their application requires careful consideration of safety implications.

However, significant challenges remain for commercial translation, including matrix-specific optimization of antimicrobial efficacy, concerns regarding nanoparticle migration and toxicity, and the lack of harmonized regulatory frameworks. Future progress will require real food validation studies, comprehensive safety assessments, development of sustainable materials, and transparent communication to ensure consumer acceptance.

Active antimicrobial packaging represents a significant advancement in food preservation, with clear potential to improve both safety and quality. Its continued development, underpinned by rigorous scientific assessment and well-defined regulatory frameworks, will be crucial for addressing the persistent global challenges of foodborne illness and food waste.

## Figures and Tables

**Table 1:** Summary of main pathogens and spoilage organisms by food category.

Food Category	Main Pathogens	Key Spoilage Microorganisms	Citations
Meat & Poultry	*Salmonella*, *L. monocytogenes*, *E. coli*	*Pseudomonas*, *Bacillus*, molds, yeasts	[1,3,8]
Dairy	*L. monocytogenes*, *S. aureus*, *E. coli*	*Pseudomonas*, *Bacillus*, lactic acid bacteria	[1,8,11]
Seafood	*Vibrio* spp., *L. monocytogenes*	*Pseudomonas*, *Shewanella*, molds	[9,10,12]
Fruits & Vegetables	*Salmonella*, *E. coli*, *L. monocytogenes*	Yeasts, molds (Aspergillus, Fusarium)	[4,12,13]
Plant-based Analogs	*Salmonella*, *L. monocytogenes*, *E. coli*	*Bacillus*, *Clostridium*, Enterobacteriaceae	[8,12]
Bakery Products	*S. aureus*, *Bacillus cereus*	Molds, yeasts	[7,12,13]

**Table 2:** Key limitations of MAP and VP.

Limitation	Description	Citations
Selective microbial inhibition	Favors LAB and anaerobes, may not suppress all pathogens	[14,15,16,17]
Temperature sensitivity	Efficacy drops with temperature abuse, risk of psychrotrophic pathogen growth	[16,17]
Packaging material permeability	Gas diffusion and package defects alter internal atmosphere, reducing microbial control	[16,19]
Incomplete spoilage prevention	Does not fully prevent enzymatic/oxidative spoilage; may affect texture and sensory quality	[15,20,21,22]
Operational and safety concerns	Requires skilled staff, high costs; risk of hazards with improper use	[16,23]

**Table 3:** Key developments in active food packaging (2014–2024).

Innovation Area	Description & Impact	Citations
Natural bioactive agents	Essential oils, plant extracts for antimicrobial/antioxidant activity	[29,32,36]
Encapsulation technologies	Controlled release, improved stability of active compounds	[26,28,29]
Biodegradable polymers	Chitosan, PLA, starch for sustainable packaging	[29,30]
Nanotechnology	Nano/microencapsulation, nanomaterials for enhanced properties	[31,32,37]
Multifunctional smart systems	Integration of active and intelligent packaging functions	[24,34]

**Table 4:** Key factors influencing microbial growth in packaged foods.

Matrix	Key Microbes	pH Influence	Water Activity (aw)	Storage/Packaging Impact	Citations
Meat	LAB, Pseudomonas, Brochothrix	Neutral pH favors bacteria	High aw accelerates growth	MAP/vacuum slows spoilage, temp critical	[12,40,41,42,43]
Fish	Pseudomonas, Carnobacterium, Serratia	Neutral pH, high protein	Very high aw, rapid spoilage	MAP/low temp extends shelf life	[15,38,39]
Dairy	LAB, spoilage bacteria	pH and protein content key	Moderate-high aw	Refrigeration, packaging essential	[12,44]
Fruits	Yeasts, molds	Acidic pH inhibits bacteria	Variable aw, higher in cut fruit	Low aw, acidity, packaging help	[12,44]

**Table 5:** Responses of spoilage microorganisms to natural antimicrobial active packaging.

Microorganism	Packaging/Antimicrobial Type	Food Matrix	Observed Response/Efficacy	Citations
*Pseudomonas* spp.	Chitosan-based, essential oils, yeast films	Oranges, tomatoes, cellulose films	Inhibition zones (10–16 mm); shelf life extension; reduced counts	[84,85,86]
*Shewanella putrefaciens*	Methoxyphenols, terpenes	In vitro	High sensitivity (IC50 ~0.35–0.75 mM); strong inhibition	[79,80]
*Brochothrix thermosphacta*	Phenyllactic acid, essential oils, nanocarriers	Pork, chicken, beef	≥4-log reduction in 1 h (nanocarriers); growth and odor inhibition; shelf life extension	[75,81,82,87]
*Photobacterium* spp.	Bacteriophage-based packaging	General foods	Targeted inhibition (reviewed as effective)	[88]
Yeasts and molds	Essential oils (lemongrass, salicylate), chitosan	Carrots, grapes, cellulose films	Strong inhibition (e.g., *Penicillium* spp. suppressed at ≥250 μL/L); shelf life extension	[83,84,85]
Mixed spoilage bacteria	Alcoholic distillate (tsipouro), polysaccharide films	Ground beef, various	2-fold shelf life extension; broad-spectrum inhibition	[89,90]

**Table 6:** Documented effects of natural antimicrobial packaging on pathogens in real foods.

Pathogen	Packaging Type/Agent	Food Matrix	Effect (Log Reduction/Outcome)	Citations
*L. monocytogenes*	Nisin, essential oils, Cu NP	Ham, cheese, meat	4–6 log reduction, complete inhibition	[91,92,93,94,95,96]
*Salmonella* spp.	Caprylic acid, diacetyl, EO	Meat, beef, cheese	1–3 log reduction, up to 77% inhibition	[88,95,96,97,98]
*E. coli* O157:H7	EO, phage, chitosan, curcumin	Beef, produce, cheese	>99% reduction, strong biofilm inhibition	[33,95,99,100,101]
Spore-formers (Bacillus)	Phage, metal NPs, chitosan	Meat, dairy	Significant inhibition	[84,88,102,103]

**Table 7:** Effects of key factors on antimicrobial packaging efficacy.

Factor	Effect on Antimicrobial Efficacy	Citations
pH	Alters release and activity; acidic pH often enhances efficacy	[44,107]
a_w_	Lower aw slows release and microbial growth; may reduce efficacy	[107,109]
Food Composition	High fat absorbs agents, reducing efficacy; protein may enhance	[88,106,110,111]
Storage Temp	Higher temp. increases release and efficacy; low temp. slows both	[102,106,107,112,113]

## Data Availability

No new data were created or analyzed in this study. Data sharing is not applicable to this article.
